# On the structural connectivity of large-scale models of brain networks at cellular level

**DOI:** 10.1038/s41598-021-83759-z

**Published:** 2021-02-23

**Authors:** Giuseppe Giacopelli, Domenico Tegolo, Emiliano Spera, Michele Migliore

**Affiliations:** 1 Department of Mathematics and Informatics, University of Palermo, Palermo, Italy; 2 Institute of Biophysics, National Research Council, Palermo, Italy

**Keywords:** Network models, Computational biophysics

## Abstract

The brain’s structural connectivity plays a fundamental role in determining how neuron networks generate, process, and transfer information within and between brain regions. The underlying mechanisms are extremely difficult to study experimentally and, in many cases, large-scale model networks are of great help. However, the implementation of these models relies on experimental findings that are often sparse and limited. Their predicting power ultimately depends on how closely a model’s connectivity represents the real system. Here we argue that the data-driven probabilistic rules, widely used to build neuronal network models, may not be appropriate to represent the dynamics of the corresponding biological system. To solve this problem, we propose to use a new mathematical framework able to use sparse and limited experimental data to quantitatively reproduce the structural connectivity of biological brain networks at cellular level.

## Introduction

The cellular connectivity of biological brain networks can be considered as the basic me chanism for information transfer in the brain. It can be represented as a graph with nodes associated with the neurons’ soma and edges representing synaptic connections. Unfortunately, our knowledge in this field is rather limited to scattered information on very few brain regions. Technical limitations have prevented so far to obtain detailed experimental evidence of how individual neurons in a large brain network are connected to each other in the different brain regions. In most cases, neuronal connectivity is studied using anatomical tracers^1^, but such specialized labeling methods have shortcomings^2^. A wealth of data can be obtained at macroscopic level using diffusion tractography^3,4^, but its resolution is not enough to determine cellular connectivity. There are a few notable exceptions for which at least a part of this information is available. For example, a connectivity matrix has been determined for: (i) all neurons composing the adult *C. elegans* nervous system^5,6^, (ii) an individual interneuron in a relatively large volume of the mouse thalamus^7^, (iii) a few hundred neurons in rats hippocampus slices^8^, (iv) a dense reconstruction of 1090 neurons from the inner plexiform layer of a mouse retina^9^, (v) a 3 million μm^3^ volume of L2/3 mouse primary visual cortex^10^, (vi) 226 neurons from the basal ganglia of a Songbird^11^, and vii) 1761 cell bodies from a Drosophila Optic Medulla^12^**.** Most computational models of neuronal networks (artificial or realistic), implemented to study brain circuits, still use either very limited experimental findings, almost exclusively restricted to data on local connectivity^13^, or follow random fixed connectivity rules that do not (even qualitatively) reproduce the major features observed experimentally^14,15^. This is a general problem, which is also present in reproducing social or real-world networks^16^.

Modeling cellular-level brain networks is directly correlated to network science, which includes different types of implementation in relation to: (i) the type of network connectivity property of the target model, (ii) the implementation strategy, (iii) the overall goal of the model. For example, network connectivity properties can be described by considering the degree distribution^17,18^, clustering^19^, small-world coupling^19^, modularity and community organization^20^, rich-club ordering^21,22^, or local community effect on connectivity growth^23^. Recent approaches, such as the non-uniform Popularity Similarity Optimization^24^ can even allow generating network topologies with quantitatively controlled levels of these properties. The implementation strategy for a network connectivity can be based on deterministic^23^ or stochastic rules, such as those mentioned above. Finally, and most relevant for the models discussed in this work, a model can suggest different implementation strategies. In our case, the main aim was to introduce a new modeling strategy, able to generate networks with degree distributions consistent with those observed among individual neurons. This is important, because the degree distributions are the major determinants of network motifs and mean field activity^25^. However, the degree distributions of most network models so far have been obtained by using constant random connection probabilities, which do not offer an appropriate mathematical description of the structural connectivity observed in vivo for cellular-level brain networks. The theoretical aspect of this issue has been introduced and discussed in previous works^26,27^, which introduced a formal mathematical representation of the experimental findings on the individual neurons’ connectivity.

Here, we show that cellular connectivity plays a major role in determining a network’s dynamics. The response of a network with structural connectivity similar to that observed in vivo can be significantly different from a network based on the random constant connection probabilities that are almost exclusively used in the computational neuroscience field. We thus suggest a new mathematical framework to describe brain networks connectivity, which can provide a better interpretation of experimental data and a better way to build large-scale brain model networks at cellular level.

## Results

### Analysis of experimentally determined networks

**Figure 1:**
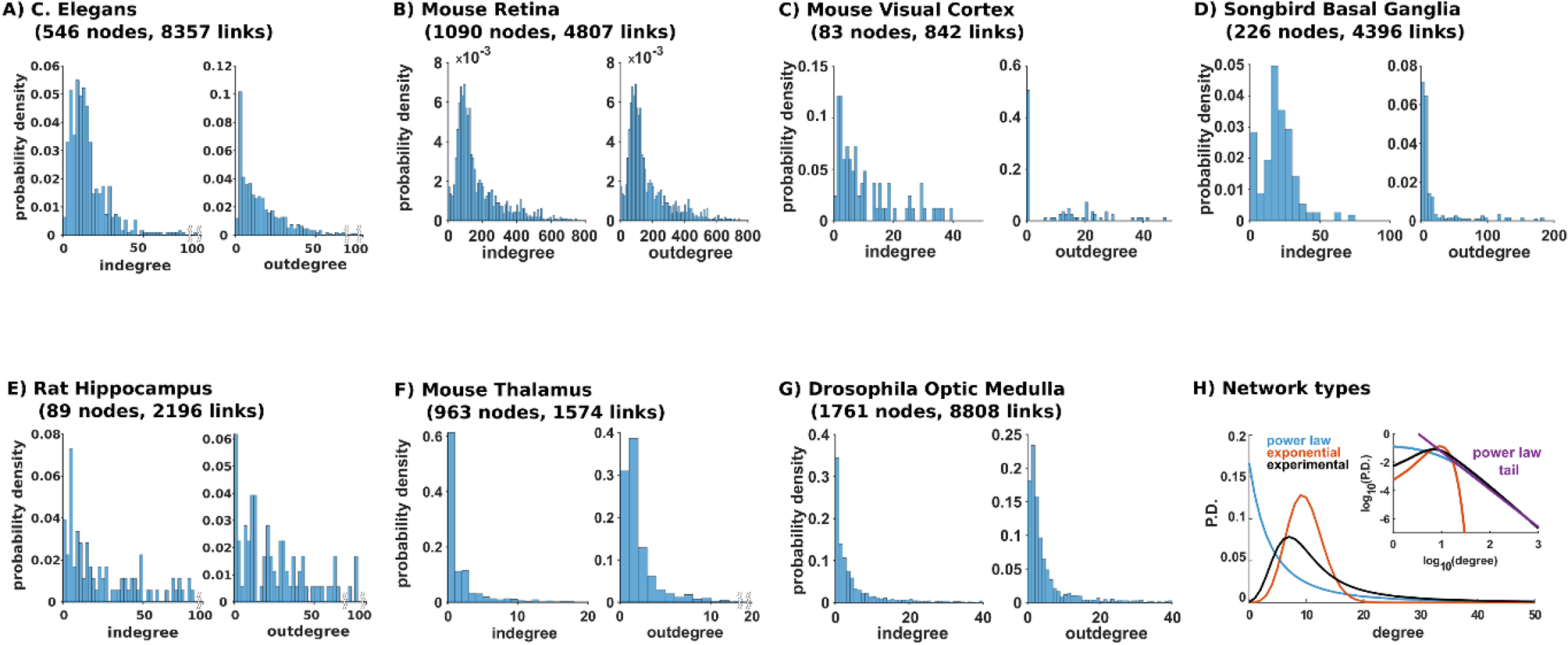
Degree distributions of different brain networks. (**A**) The 546 neurons of the *C. elegans* adult male nervous system^5^; (**B**) from a dense reconstruction of 1090 neurons from a mouse retina inner plexiform layer^9^; (**C**) from electron microscopy data on a 3 million μm^3^ volume of L2/3 mouse primary visual corte^10^; (**D)** from electron microscopy data on 226 neurons from a Songbird basal ganglia^11^; (**E**) 89 neurons from a slice from a rodent hippocampus^8^ (courtesy of Paolo Bonifazi); (**F**) mouse thalamus (Morgan et al.^7^); (**G**) from electron microscopy data containing 1761 body ID’s from a Drosophila Optic Medulla^12^; (**H**) Schematic representation of degree distribution for different model networks: Power Law (blue), exponential (red) and stereotypical experimentally observed distribution (black). The inset shows a log–log plot with a (purple) line representing the Power Law tail; note how the power law tail line fails to reproduce the low degree connectivity.

We started by considering typical experimental findings for brain network connectivity at cellular level. In Fig. 1 we show the in- and out-degrees calculated from different brain regions. For the *C. elegans* brain^5^ (Fig. 1A), the degree distributions closely follow what is expected for full brain networks: a small value for low-degrees (i.e. no disconnected or poorly connected neurons), rapidly increasing to a peak indicating the most likely number of connections, and a more or less slow decrease to zero for higher degrees (i.e. there are no neurons connected to all the other neurons). Essentially the same type of distribution was found for the inner plexiform layer of the mouse retina^9^ (Fig. 1B) and visual cortex^10^ (Fig. 1C). For other brain regions, such as the Songbird Basal Ganglia^11^ (Fig. 1D), the mouse hippocampus^8^ (Fig. 1E) and thalamus^7^ (Fig. 1F), and for the Optic Medulla of a Drosophila^12^ (Fig. 1G), the degree distributions exhibited a significant proportion of disconnected or poorly connected cells, most likely caused by the finite size of the network obtained after the cutting procedure.

Until recently, there was no mathematical description of these types of distribution. A rigorous theoretical description is the only way to guarantee that a brain model network is consistent with the experimentally observed main connectivity. This situation is schematically illustrated in Fig. 1D, where we plotted a stereotypical example of (in- or out-) degree distribution for a biological brain network (Fig. 1D, black curve), together with the distributions obtained from two classic types of theoretical network model: power law (Fig. 1D, red, Erdős curve^17^) and exponential (Fig. 1D, blue, Barabási curve^18^). Since a power law model can be represented by the tail of its log–log plot of degree distribution (see Fig. 1D purple line in the inset), a very common choice is to analyze the brain structural connectivity considering only this property. The problem with this approach is that it ignores a large part of the connectivity range, and thus it would create a network with a large and unrealistic proportion of neurons with low connectivity. For this reason, another very popular method to implement a network is to generate an exponential connectivity model. Also in this case, the corresponding network may not appropriately represent the connectivity of a biological network, because it will have too few hub neurons. It should be noted that different brain regions might be organized according to connectivity paradigms that may not follow the expected distributions discussed here, i.e. with an exponential distribution for low degrees and a power-law tail for high degrees. For instance, the olfactory bulb is known to have a very different organization. However, taken together, these results suggest that the connectivity of real brain networks does not follow the commonly adopted rules to formulate most of the neuronal network models. In the next paragraphs, we will show the possible consequences for the network dynamics in a few cases.

### Analysis of computational model networks

**Figure 2:**
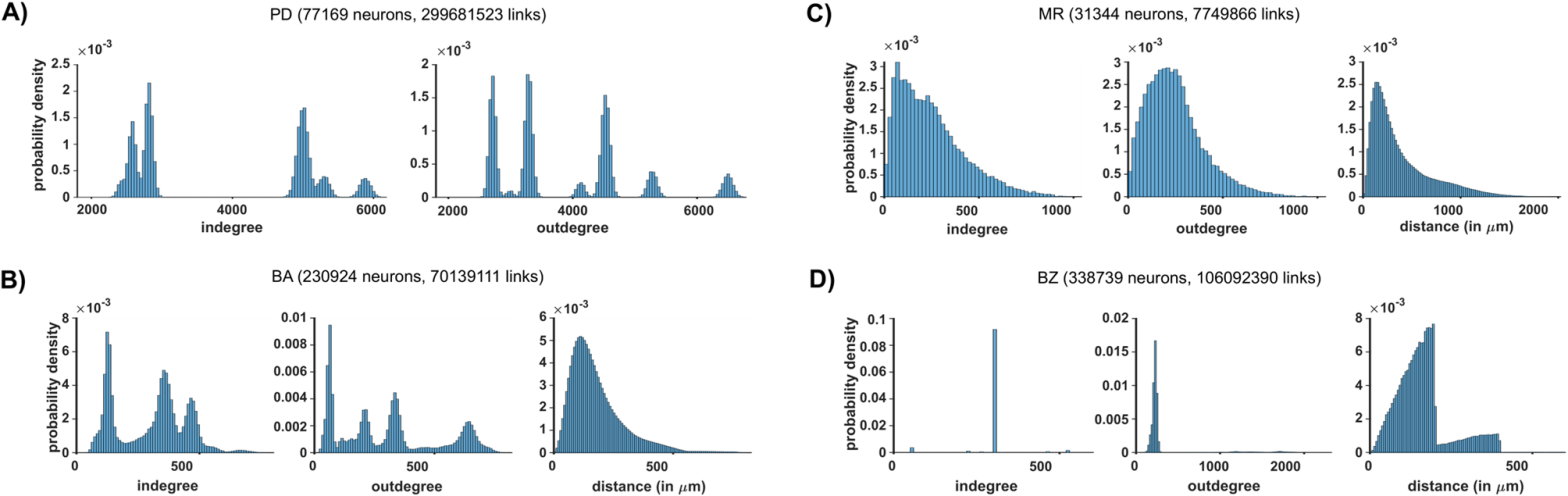
Degree distributions of different large-scale models and connection length distribution. (**A**) Degree distributions of a 1 mm^2^ cortical column model^33^, indicated as PD; connection lengths are not available. (**B**) Degree distributions and connection length distribution of a visual cortex model^29^, indicated as BA. (**C**) Degree distributions and connection length distribution of a neocortical column model^30^. (**D**) Degree distributions and connection length distribution of a hippocampus CA1 area model^13^, indicated as BZ.

To better illustrate the difference among the common rules used to build model networks, we considered the connectivity of four published large-scale models of brain regions (Fig. 2). It should be stressed that all of them were based on experimental findings obtained in vitro*,* since in vivo studies of connectivity at the cellular level are not feasible. Some properties of real brain networks may thus still be missing. However, this issue was outside the scope of this work, and it has not been investigated further. In Fig. 2A we show the degree distributions of a spiking neuron network model (indicated as PD in the rest of the paper) of a cortical microcircuit based on experimental data obtained in vitro^1,28^. We calculated its connectivity directly from the published code. It is a combination of Gaussian distributions, typical of exponential subnetworks (see the red curve in Fig. 1D) with a constant and distance-independent connection probability between any two given neuron populations. Figure 2B shows the distributions of a large-scale visual cortex model^29^ (indicated as PD in the rest of the paper) with neurons distributed in a 3D space, as in the real system. In this case, the network was implemented using an exponential function that included distance-dependent information. In Fig. 2C, we show the connectivity of a neocortical model^30^ (indicated as MR in the rest of the paper), implemented from fully reconstructed morphologies, distributed in a 3D space, connected using a complex empirical approach including touch-detection, pruning procedures, and distance-dependent information^28^. In this case, the resulting degree distributions have all the features observed experimentally. A similar approach was also followed in building a full-scale model of the olfactory bulb^31^ and of the striatum^32^. Finally, in Fig. 2D we plot the distributions for a full-scale model of a rodent hippocampal CA1 region^13^ (indicated as BZ in the rest of the paper), implemented using full (identical) morphologies laid out on a planar surface. It exhibits an exponential connectivity, with a distance-dependence that is shaped by the planar architecture. These results show that brain network models, connected using an exponential random network rule, generate an overall structural connectivity that is dramatically different from that observed experimentally^10^.

### Functional tests using the same model with different connectivity

It is relevant to note that each model analyzed in the previous subsections has been originally implemented to investigate a specific topic, and validated against a specific set of experimental findings. For example, the two models built using purely exponential random connectivity, the PD^33^ and BZ^13^, reproduced the cortical cell-type specific firing recorded in vivo in awake animals, and the preferential firing of distinct interneuron types during different phases of theta oscillations, respectively. It is outside the scope of this work to investigate if these (or other) model networks would behave in a different way, if connected following the real connectivity observed in the corresponding brain regions. However, we believe that it is interesting to see how the same network responds to the same input when connected using different rules, including one able to reproduce the typical degree connectivity observed in real brain systems.

For this purpose, we carried out test simulations with a reference model network, composed of 12,500 neurons implemented as a balanced network of excitatory (80%) and inhibitory (20%) neurons, with an integrate-and-fire spiking behavior and randomly connected with a fixed 0.1 probability. We will call this model BR. The excitatory/inhibitory proportion is consistent with that found in many brain areas, and similar to that used for other cortical models. The BR model^14,34^ was downloaded from the NEST^15^ website (www.nest-simulator.org/py_sample/brunel_alpha_nest/). To test different connectivity rules, we built two additional models, by extending the BR model to 30,000 neurons (25,000 excitatory and 5,000 inhibitory), and connecting them following Potjans and Diesmann model^33^ (Fig. 2A, indicated as BR-PD in the rest of the paper) or as in Markram et al^30^ model (Fig. 2C, indicated as BR-MR in the rest of the paper), keeping all the cellular and synaptic properties the same as in the original BR model. We have preferred to use the reference model *as is*, with a number of neurons (12,500 cells) lower than those composing the PD and MR models (approximately 75,000 and 30,000 cells, respectively). We do not expect any difference using a larger network, given the relatively high number of neurons and the constant connection probability.

**Figure 3:**
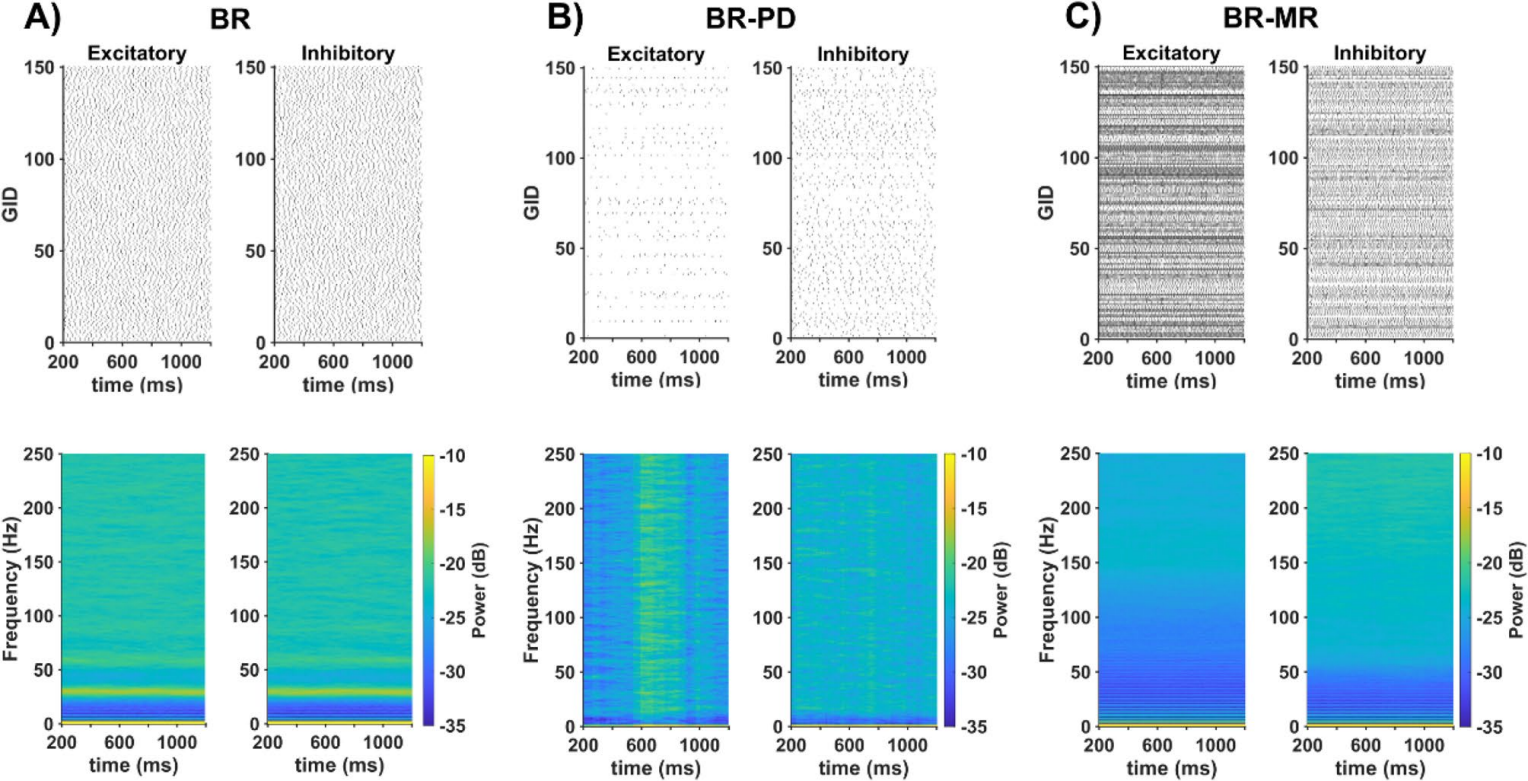
Network behaviors for different connectivity. Panels illustrate the raster plots of 150 randomly chosen neurons denoted by their GID (a unique number associated to each neuron) (top), and the corresponding power spectra (bottom) during a simulation with the same random background activity. (**A**) A 12,500-neuron network connected as in BR^14^, (**B**) connected as in PD^33^ with 30,000 neurons, (**C**) connected as in MR^30^ with 30,000 neurons.

To reproduce in the BR-PD model the same random connectivity of the PD model^33^, we used the same equations described in the original paper^33^. For the BR-MR model, we directly used the original connectivity matrix of the MR model^30^, available in the Neocortical Microcircuit Portal (https://bbpteam.epfl.ch/nmc-portal/welcome.html). We then tested the behaviors of each network, by running 1.4 s simulations in which all neurons were randomly and independently activated with a Poisson input with an average frequency of 17 Hz. For the reference model, this corresponded to a frequency twofold higher than the threshold value (see Fig. 8C in Brunel et al.^14^). A raster plot from 150 randomly chosen excitatory and inhibitory neurons and the power spectra of their activity during 1 s of simulation (from *t* = 200 to *t* = 1200 ms) are shown in Fig. 3A. The plots highlight a very similar activity for both populations, a consequence of the uniform and constant connectivity; the average global frequency has a main component at 26 Hz and progressively weaker harmonics. For the BR-PD model (see Fig. 3B), the raster plots show a significant difference in the overall activity between excitatory and inhibitory neurons (much lower for excitatory neurons), and also a large firing variability among neurons of the same type. This resulted in rather featureless power spectra. The BR-MR model (see Fig. 2C), exhibited a wider range of firing patterns, in comparison with the other two models, because the input elicited a much stronger activity; the essentially asynchronous and high-frequency firing patterns resulted in power spectra weaker for lower frequencies. These results already suggest a clear qualitative difference between the response of a model connected using a uniform random connectivity, such as in Brunel et al.^14^, and models using a more diversified rule as in PD^33^ or MR^30^.

**Figure 4:**
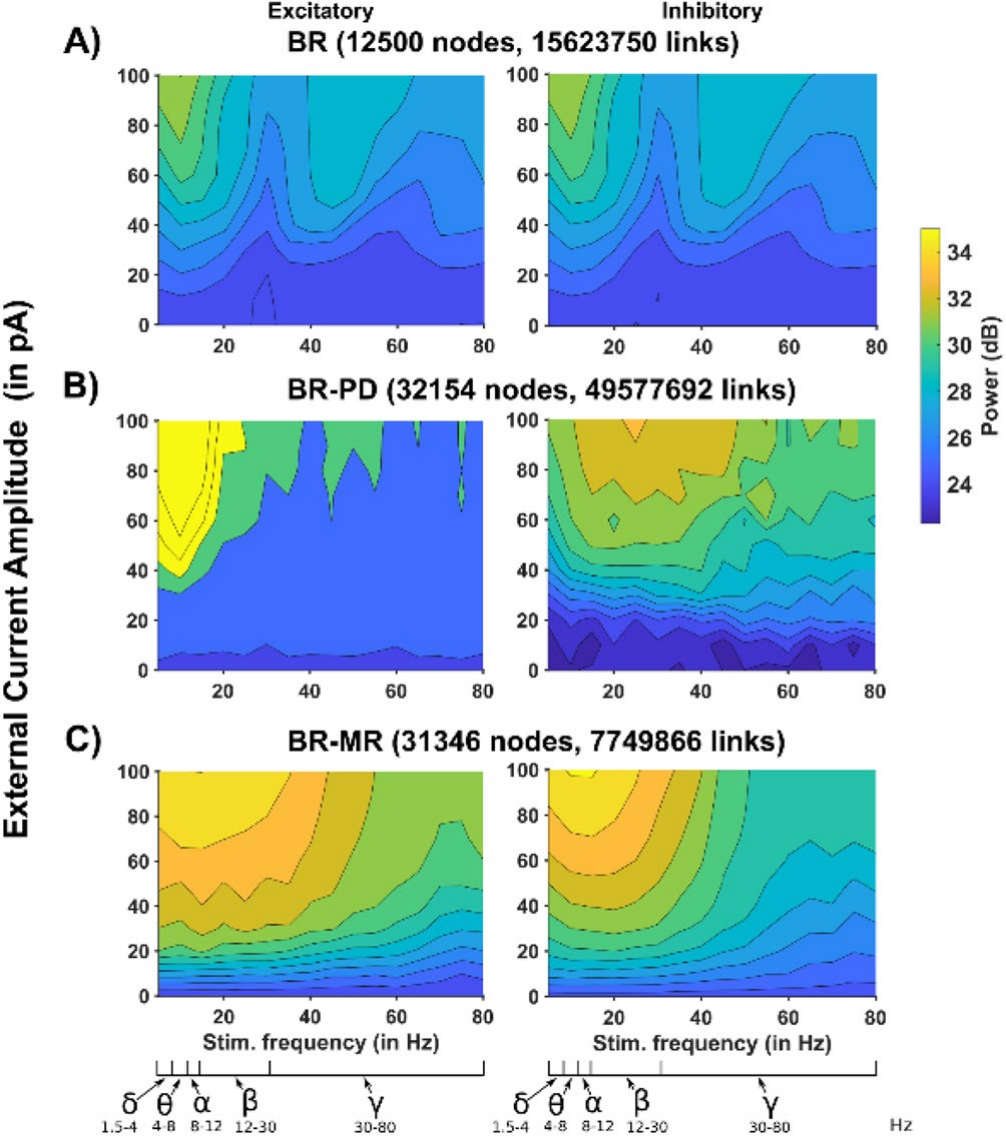
Power spectra as a function of external stimulation amplitude and frequency. (Left) Excitatory neurons, (Right) Inhibitory neurons. (**A**) A 12,500-neuron network connected as in BR^14,34^, (**B**) connected as in PD^33^ with 30,000 neurons, (**C**) connected as in MR^30^ with 30,000 neurons. Major brain rhythms frequency range (from Buzsáki and Draguh^35^) are shown below the stimulation frequency axis.

**Figure 5:**
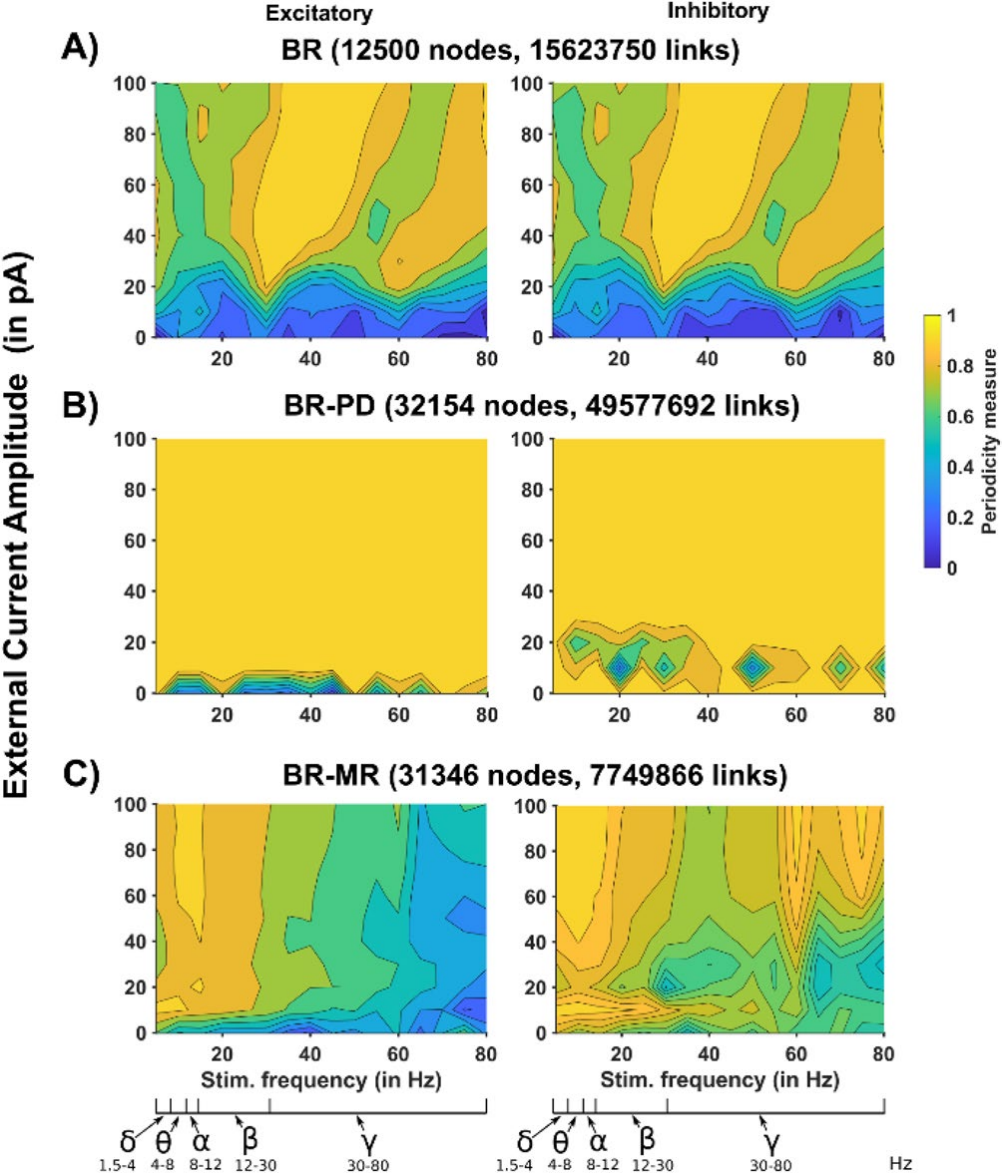
Cosine distance as a function of external stimulation amplitude and frequency. (Left) Excitatory neurons, (Right) Inhibitory neurons. (**A**) A 12,500-neuron network connected as in BR^14,34^, (**B**) connected as in PD^33^ with 30,000 neurons, (**C**) connected as in MR^30^ with 30,000 neurons. Major brain rhythm ranges (from Buzsáki and Draguhn^35^) are shown below the stimulation frequency axis.

To have a more complete overall picture of how the different connectivity rules can change the response of a network to oscillatory inputs, we carried out a set of simulations for each model. In each simulation, we used a synchronized sinusoidal input current to all neurons, and calculated the change in the power spectrum, with respect to the random background. The results, calculated from the average activity of 150 randomly chosen neurons during a 1 s time window, are shown in Fig. 4 as function of the external current amplitude and frequency. For the BR model (Fig. 4A) we found an essentially identical response for excitatory and inhibitory neurons, as observed for random background noise (see Fig. 3). In the range of the tested current amplitude (0–100 pA), the additional sinusoidal input did not appear to have a significant impact on the global network oscillatory activity. As function of the frequency, the largest response was in the delta and theta range, less pronounced in the low gamma, and weaker in the beta and high gamma range. With the BR-PD model (Fig. 4B), the excitatory neurons population exhibited a strong effect limited to the low frequency rhythms (Delta to Alpha), a relatively much weaker response in the Beta range and essentially unresponsive to higher frequencies, even with strong inputs currents. The inhibitory population was more responsive in the relatively wide range of frequencies corresponding to the Alpha-low Gamma range. The BR-MR model (Fig. 4C) had an overall smoother response that was more pronounced for excitatory neurons in a wide range of frequencies covering the Delta to low Gamma bands, and progressively lower for higher frequencies. A similar response was observed for the inhibitory population, although limited to the Delta-Beta range. These results suggest that different connectivity profiles can significantly modulate a network response to oscillatory inputs. nother crucial metric, to determine how a network responds to an oscillatory input, is the cosine distance. It gives a direct measure of how well the network activity follows an oscillatory input, and it is a measure of synchrony rather than power. We thus calculated, from the same set of simulation carried out for Fig. 4, the cosine distance of each model network as a function of input current amplitude and frequency. The results are shown in Fig. 5. BR network (Fig. 5A) was able to better follow a sinusoidal input in the Beta band, progressively shifting into Gamma range for higher current amplitudes. Again, almost exactly the same response was observed for the inhibitory neuron population. The BR-PD network (Fig. 5B) was highly synchronized with essentially any external input, in the entire range of current amplitude and frequency that was tested. In the BR-MR network (Fig. 5C), the excitatory neuron population exhibited a highly synchronous response in the Delta-Beta range, which was essentially independent from the current amplitude, whereas the inhibitory population had an additional range for synchronization in the high Gamma range. Taken together these results demonstrate that cellular connectivity plays a major role in determining how a network will follow an oscillatory input, and that the response of a network with a structural connectivity similar to that observed in vivo may be significantly different from a network based on constant random probabilities.

### A new network connectivity model

The question now is which connectivity should be used to build a network aiming at reproducing the dynamics of a real brain network. Ideally, it should have the same connectivity properties as the corresponding biological system, because we have shown that different types of connectivity may cause the network to respond in a quite different way. Unfortunately, except for a few rare cases, the real cellular connectivity is not available with sufficient details. However, as discussed for Fig. 1H, we can expect that the degree distributions of a real brain network can be approximated by the empirical connectivity rules used, for example, in the MR model. The problem is that it can be obtained only by adopting somewhat ad-hoc touch detection and pruning procedures and that, most importantly, they always require a full-scale model implementation including dendritic and axonal trees.

**Figure 6:**
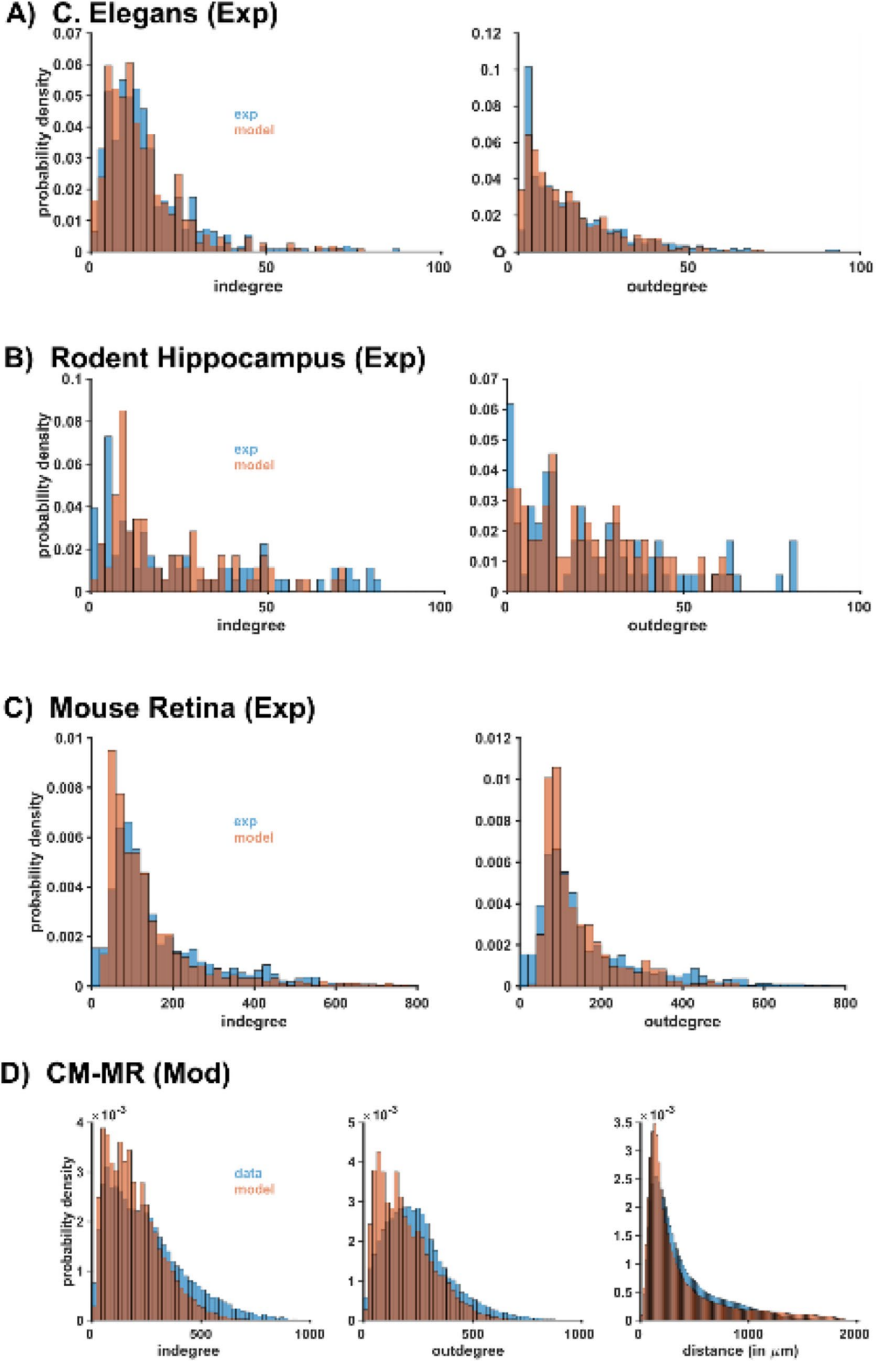
Fitting the degree distributions observed in different brain networks. In all cases the model (orange bars) represent the fit of the experimental findings (blue bars) using the algorithm described in Giacopelli et al.^26^ complemented, when available, with distance-dependent information^27^. The parameters used for each case are (see “Methods” for symbols): (**A**) two blocks configuration with δ = 1.5 ± 0.5, E_k_ = 1 ± 0.5, L = 1, η = 3 ± 0.25, φ_u_ = 1 and φ_d_ = 0 ± 0.00001; (**B**) Two blocks asymmetric configuration with δ = 0 ± 2, E_k_ = 0.1 ± 0.05, L = 1, η = 3 ± 0.25, φ_u_ = 0.75 and φ_d_ = 0 ± 0.001; (**C**) Two blocks (with one block inverted) configuration with δ = 0 ± 0.5, E_k_ = 70 ± 0.05, L = 3 ± 0.75, η = 3 ± 0.25, φ_u_ = 1 and φ_d_ = 0 ± 0.001; (**D**) The model is composed by 4 components with a 6-layers structure each composed by 9 blocks: Excitatory-Excitatory component, with parameters δ = 3 ± 0.5, E_k_ = 100 ± 0.5, L = 100 ± 0.5, η = 3 ± 0.25, φ_u_ = 1 and φ_d_ = 0.0001 ± 0.00001; Excitatory-Inhibitory component, with parameters δ = 4.52 ± 1, E_k_ = 6.8 ± 0.5, L = 6 ± 1, η = 2.56 ± 0.5, φ_u_ = 1 and φ_d_ = 0.0003 ± 0.00025; Inhibitory-Inhibitory component, with parameters δ = 1 ± 0.5, E_k_ = 3 ± 0.5, L = 4 ± 1, η = 3 ± 0.25, φ_u_ = 1 and φ_d_ = 0.0001 ± 0.00001; Inhibitory-Excitatory component, with parameters δ = 3.27 ± 1, E_k_ = 7.05 ± 0.5, L = 5 ± 1, η = 3.66 ± 0.5, φ_u_ = 1 and φ_d_ = 0.0006 ± 0.00025.

An alternative way is to use a recent theoretical framework^26,27^ demonstrating that any network, with any biologically plausible degree and connection length distribution, can be algorithmically reproduced by an appropriate convolution of an exponential and a power-law model. Such a methodology allows creating a network with the desired degree distributions, in quantitative agreement with the available experimental findings, without the need for a detailed implementation of the full-scale system. The theoretical background and the implementation details are reported in “Methods” (see “Convolutive connectivity model” section). Although this approach does not ensure that the model and experimental networks will be equivalent in a graph-theoretical sense, having the same degree distributions guarantees that at least the network motifs and mean-field activity will also be the same^25^. To demonstrate the flexibility of this new connectivity model, in Fig. 6 we compared the degree distributions obtained by fitting the distributions observed in a *C. elegans* brain network (Fig. 6A), in the mouse hippocampus (Fig. 6B) and retina (Fig. 6C), and in the MR network (Fig. 6D). In all cases, we were able to find a set of model parameters resulting in a network statistically indistinguishable from the original (See Methods for *p*-value calculation). For the *C. elegans* model, the null hypothesis was validated 80% of the times, about 70% for MR model, approximately 85% of the times for the hippocampal slice model, and approximately 100% of the times for the retina model. Note that, when available (as for MR model), the algorithm was also able to take the connection length distribution into account. These results point out to a new way to build brain networks, using an algorithm able to capture the essential features of a biological neuron network, instead of using less reliable random, constant probability, connectivity schemes.

**Figure 7:**
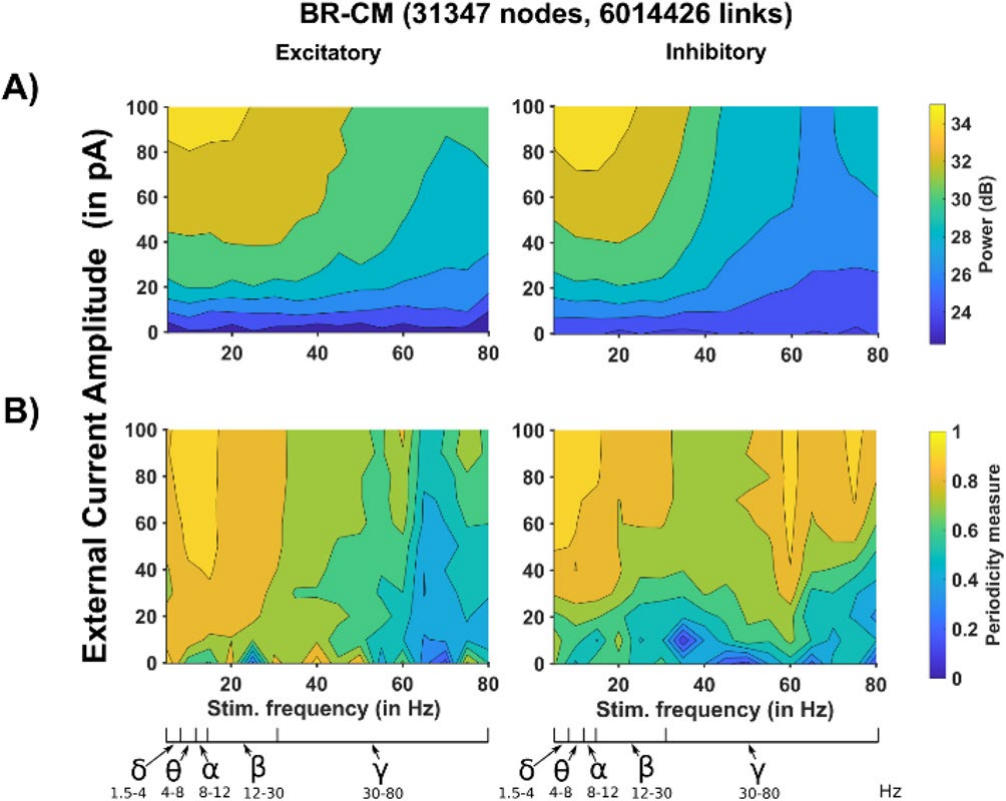
Network activity using the Convolutive Model connectivity. (**A**) Power spectra as a function of external stimulation amplitude and frequency of Excitatory (Left) and Inhibitory (Right) neurons for the Convolutive model^26^ fitting the MR network^30^; (**B**) Cosine distance as a function of external stimulation amplitude and frequency of Excitatory (Left) and Inhibitory (Right) neurons for the Convolutive model^26^ fitting the MR network^30^.

To test how a network, based on our connectivity model, behaves in terms of power spectra and cosine distance, we run a set of simulations using the distributions obtained by fitting those of the MR model (orange bars in Fig. 6D, indicated as CM-MR). Given the extensive validation of the MR model against a series of experimental findings^28,30^, it appears to be the best implementation of a biological brain network connectivity. The same comparison with, for example, the PD model would not be appropriate for two reasons: (1) its connectivity is far from what expected for a real network (compare the distribution in Fig. 2A with those in Fig. 1), and (2) it is not clear which real connectivity the model intended to implement. The plots in Fig. 7 demonstrate that the results are in very good agreement with those obtained with the original MR model connectivity (Figs. 4C and 5C). These results suggest that our approach can be a valid alternative to the empirical, time consuming, and full morphologies-dependent touch detection procedures, to implement the type of connectivity observed in brain networks.

## Discussion

One of the major goals of this work was to point out to the community that the difference in the structural connectivity between biological and computational brain networks, can have substantial functional consequences in terms of how a network will respond to an external input. Sensorial inputs or internal brain activity elicit a flurry of activity in different brain regions at different times and under different conditions, which are ultimately responsible for the emergence of macroscopic synchronized oscillatory rhythms at different frequencies^35^.

The specific connectivity of different brain areas (together with intrinsic neuronal and synaptic properties) can be a major determinant in shaping these oscillations, and a computational model network should be implemented taking this into account. We think that this is a very important point, and it poses the question of how to implement a model network with the appropriate structural connectivity. As we have shown, exponential models, which are relatively straightforward to implement (e.g. BR^14,34^, PD^33^, Bezaire et al*.*^13^, Izhikevich and Edelman^36^), do not reproduce the experimentally observed degree distributions, although they are based on selected experimental findings. The major problem of this type of implementation is the use of a constant and distance-independent connectivity. A data driven approach, following touch detection and pruning schemes (such as in MR^30^ and Hjorth et al.^32^), is able to generate the most accurate representation of a biological network connectivity, but these procedures require very detailed experimental constrains on axonal and dendritic arborization and supercomputing resources, especially for full-scale networks.

This problem can be avoided using our approach that, to the best of our knowledge, is the only one available in the field so far. It is based on a rigorous theoretical framework, and it is able not only to reproduce any given brain network conectivity, starting from limited experimental information on in- and out-degrees, but it can also interpret the network connectomic in terms of an appropriate combination of exponential and power law components. The mathematical framework guarantees that the degree distributions have the properties expected for a biological brain network, i.e. an exponential distribution for low degrees and a power-law tail for high degrees, and the corresponding algorithm can be adapted to fit any experimentally observed degree distribution. In order to make the model more attractive and easier its use for modelers and interest readers, a web application has been made available online (see the paragraph on “Convolutive connectivity model” in “Methods”), to build a cellular level connectivity matrix starting from the desired degree and connection length distributions. It should be noted that, in this paper, we were not interested in comparing different published models per se but, rather, in using their respective connectivity with the same reference model, to show that different connectivity rules can give different results. The overall conclusions of this paper can be summarized as follows:

1. The random connection probabilities models are the simplest and most used connectivity models. They have the advantage to require just the probability of connection between populations to connect a network, and they are fast in terms of implementation time. A negative aspect is that they poorly match the degree distributions observed experimentally, leading to a potential activity mismatch with the corresponding real network they aim to implement.
2. Connectivity models based on touch detection are the most realistic models available. Their main problems are that they require a massive amount of experimental morphological and structural constrains and large computational resources.
3. The Convolutive model used in this work can reproduce the full degree distributions experimentally observed using sparse experimental data and a much lower computational time to build the network. The drawback is that the mode ignores additional topological and intrinsic properties that may further modulate a network’s behavior. For example, the Convolutive model so far does not consider absolute synaptic weights and multiple connections between any two given neurons. We will consider the theoretical and practical consequences of these additional characteristics when sufficient experimental constraints on their distributions will be available.

In conclusion, this work should be considered as a first step into the problem of providing a better way to build in silico networks able to reproduce the experimentally observed degree distributions, which are the only determinants of network motifs and mean field activity^25^. We thus suggest to use this framework to implement or analyze the structural cellular connectivity of large-scale biological brain networks.

## Methods

### Model network and simulations

Simulations were carried out using NEST^15^ (www.nest-simulator.org). All models and simulation files will be available in the ModelDB database (https://senselab.med.yale.edu/modeldb/, acc.n.266506). Additional material and a web application will also be available in the “Live papers” section of the Human Brain Project Brain Simulation Platform https://humanbrainproject.github.io/hbp-bsp-live-papers/index.html.

For all simulations we used a model downloaded from the NEST simulator website (www.nest-simulator.org/py_sample/brunel_alpha_nest/) as a reference. It was introduced in BR^14,34^, and it was implemented as a network of leaky Integrate and Fire (LIF) neurons. The membrane potential for each neuron in the network was calculated as:

$$\tau \dot{{V}_{h}}\left(t\right)=-{V}_{h}\left(t\right)+\tau \sum_{j=1}^{N}{J}_{hj}\sum_{k}\delta (t-({t}_{j}^{k}+D))$$

where *V*_*h*_*(t)* is the membrane potential at time *t* for neuron *h*, *J*_*hj*_ = 0 if there is no connection from cell *j* to cell *h*, and *J*_*hj*_ =  ± *gJ* for inhibitory or excitatory connections, with *g* and J representing the inhibitory or excitatory synaptic current strength; *δ(t)* is the Dirac’s delta distribution, *t*_*j*_^*k*^ is the spike time of neuron *j*, which is propagated to neuron *h* with a delay *D*. All neurons are also connected to a Poisson background noise generator. In a series of simulations, all neurons were connected to the same sinusoidal current generator. In this case, the total current on each neuron was described by:

$${I}_{h}\left(t\right)= \frac{\tau }{R}\sum_{j=1}^{N}{J}_{hj}\sum_{k}\delta \left(t-\left({t}_{j}^{k}+D\right)\right)+A \mathrm{sin}(2\pi ft)$$

where *f* is the frequency (in Hz) and *A* the amplitude (in pA). For each model studied in this work, only the connectivity rules were changed. All other cell properties and stimulating protocols were the same.

### Power spectrum

To calculate the power spectrum from each simulation, we followed the approach described by Dummer et al*.*^37^. We started from the set of spike times of 150 randomly chosen excitatory and inhibitory neurons:

$${x}_{h}\left(t\right)=\sum_{k}\delta (t-{t}_{h}^{k})$$

We then considered time windows of size *T* to calculate the Short time Fourier Transform centered in *t*_*0*_

$${\stackrel{\sim }{x}}_{h}\left(\omega ,{t}_{0}\right)={\int }_{{t}_{0}-\frac{T}{2}}^{{t}_{0}+\frac{T}{2}}{e}^{2\pi i\omega \tau }{x}_{h}\left(\tau \right)d\tau =\sum_{k| \left|{t}_{h}^{k}-{t}_{0}\right| < T/2}{e}^{2\pi i\omega {t}_{h}^{k}}$$

where *h* is the imaginary unit (this symbol has been chosen to avoid confusion with notations). The modulus inside the time windows was calculated as in Dummer et al*.*^37^.

$${S}_{h}\left(\omega ,{t}_{0}\right)=\frac{\sqrt{{\stackrel{\sim }{x}}_{h}\left(\omega ,{t}_{0}\right){{\stackrel{\sim }{x}}_{h}\left(\omega ,{t}_{0}\right)}^{*}}}{T}$$

z^*^ is the conjugated of the complex number *z*. The contribution from all neurons was averaged within each time window *T* and plotted (e.g. Figure 3) as function of frequency and time as

$$L\left(\omega ,{t}_{0}\right)=\frac{\sum_{h=1}^{M}{log}_{10}({\mathrm{max}(S}_{h}\left(\omega ,{t}_{0}\right),{10}^{-4}))}{M}.$$

### Functional measures

For all simulations with an additional external sinusoidal stimulus, we plotted the results for a network stimulated with a frequency *f* and an Amplitude *A* (defined as *L*_*S*_^*(f,A)*^*(ω, t*_*0*_*)*) as the difference with respect to the control network (defined as *L*_*B*_*(ω, t*_*0*_*)):*

$${\Delta }_{S}^{\left(f,A\right)}\left(\omega ,{t}_{0}\right)={L}_{S}^{(f,A)}\left(\omega ,{t}_{0}\right)-{L}_{B}\left(\omega ,{t}_{0}\right)$$

Averaged over time as

$${D}_{S}^{\left(f,A\right)}\left(\omega \right)={\langle {\Delta }_{S}^{\left(f,A\right)}\left(\omega ,{t}_{0}\right)\rangle }_{{t}_{0}}.$$

Following Parseval’s Theorem, the power of the response in dB was calculated as

$${R}_{S}^{(f,A)}={\int }_{0}^{W}{10}^{2 {D}_{S}^{\left(f,A\right)}\left(\omega \right)}d\omega $$

where *W* is the highest harmonic that was considered (250 Hz).

The periodicity measure was calculated starting from a vector of discretized frequencies *w*_*k*_. The range [*0,W*] was subdivided in *Q* bands of width *f* and every band subdivided into *Q* partitions *{w*_*k*_^*p*^*}*_*k*=*1,…,S*_ for each *p* = *1,…,Q*. For each pair of partitions *(p, p* + *1)* the cosine distance was calculated as

$${C}_{p}^{(f,A)}=\frac{\sum_{j=1}^{S}{D}_{S}^{\left(f,A\right)}\left({w}_{j}^{p}\right){D}_{S}^{\left(f,A\right)}\left({w}_{j}^{p+1}\right)}{\sqrt{\sum_{j=1}^{S}{{D}_{S}^{\left(f,A\right)}\left({w}_{j}^{p}\right)}^{2}}\sqrt{\sum_{j=1}^{S}{{D}_{S}^{\left(f,A\right)}\left({w}_{j}^{p+1}\right)}^{2}}}$$

and averaged over *S*

$${C}^{(f,A)}=\frac{\sum_{p=1}^{Q-1}{C}_{p}^{(f,A)}}{Q-1}$$

to obtain a single number representing the periodicity of the spectrum for an oscillatory input of amplitude *A* and frequency *f*.

### Convolutive connectivity model

The connectivity model used to implement the different networks in Fig. 6 has been previously published (see Giacopelli et al*.*^26,27^). It is a convolutive model where we mixed power law and exponential connectivity to reproduce or build any given degree and connection length distributions. Briefly, to create a network *B*, the network is divided in *N*_*B*_ arbitrary blocks. Inside each block neurons are connected with a power law model minimizing the cost function

$${C}_{hj}=\frac{\delta ({d}_{hj}+{S}_{N}\eta {r}_{j})}{{S}_{F}}+{\lambda }_{j}$$

where *δ* and *η* are parameters of the model, *d*_*hj*_ is the square of Euclidean distance between the nodes *i* and *j, h*_*j*_ is the graph theoretical measure of centrality for node *j, r*_*j*_ is a random vector with uniform distribution in [0,1], *S*_*N*_ = 200μm^2^ and *S*_*F*_ = 1μm^2^ are fixed scale factors. The connections of neurons between blocks are determined by the scheme illustrated in Giacopelli et al*.*^26^, where each block is subdivided in partitions of *L* elements, and the connection patterns determined by the expected value of the kernel and two probabilities distributions *ϕ*_*u*_ and *ϕ*_*d*_. The overall scheme is a convolutive model^26^ with in- and out-degree distributions determined as:

$$P\left({D}_{B}^{I}=k\right)={\left[{f}_{q}^{I}*{\left(\left(\left(1-p\right){\delta }_{0}\left(q\right)+p{B}_{{\phi }_{u}}^{l}(q)\right)*\left(p{\delta }_{0}\left(q\right)+(1-p){B}_{{\phi }_{d}}^{l}(q)\right)\right)}^{*\left({N}_{B}-1\right)M}\right]}_{k}$$

$$P\left({D}_{B}^{O}=k\right)={\left[{f}_{q}^{O}*{\left(\left(\left(1-p\right){\delta }_{0}\left(q\right)+p{B}_{{\phi }_{u}}^{l}(q)\right)*\left(p{\delta }_{0}\left(q\right)+(1-p){B}_{{\phi }_{d}}^{l}(q)\right)\right)}^{*\left({N}_{B}-1\right)M}\right]}_{k}$$

where *δ*_*0*_*(q)* is the Dirac’s delta distribution, *B*_*p*_^*N*^*(q)* is the Binomial distribution, and *p, f*_*k*_^*I*^ and *f*_*k*_^*O*^ are model fitting parameters. The code and an online interactive application to build a given network, starting from an arbitrary set of desired degree and connection length distributions, is available in the “Live papers” section of the Human Brain Project Brain Simulation Platform https://www.humanbrainproject.eu/en/brain-simulation/live-papers/.

### P-value histogram calculation and validation

The *p*-value is a well-known method used to validate the null hypothesis, *H*, that two distributions are indistinguishable. The usual procedure to compute the *p*-value for data with nonparametric statistics is the Kolmogorov–Smirnov (KS) test, which assumes the Kolmogorov distance^38^ as a statistical measure

$$d\left({f}_{1},{f}_{2}\right)=\underset{x}{\mathrm{max}}|{F}_{1}\left(x\right)-{F}_{2}(x)|$$

where *f*_*1*_ and *f*_*2*_ are two probability density functions (PDF) and *F*_*1*_ and *F*_*2*_ are their cumulative density functions (CDF). However, our degree distributions do not follow a classic statistical model. To obtain a *p*-value estimate we have thus used a method based on the *p*-value histogram^39^, calculating a set of *p*-values as

$$ p_{i} = 2 \, \min \left\{ {P\left( {D \ge d_{0i} } \right),\;P\left( {D \le d_{0i} } \right)} \right\} $$

where *D* is an empirical random variable, under the assumption of *H*, and

$${d}_{0i}=d\left({f}_{0},{f}_{i}\right), with i=1,\dots ,n$$

are the distances calculated from the reference distribution, *f*_*0*_, and the distributions, *f*_*i*_, obtained from *n* instances of our model. Then we calculated the histogram of *p*_*i*_ with bin α = 0.05 and considered the number of elements in the first bin. Under these conditions, if the null hypothesis is true, *p*_*i*_ should be higher than a 0.05 threshold approximately 95% of the times. This method has been separately applied to the degree distributions and to the connection length distributions.
